# Thermal and Flammability Analysis of Polyurethane Foams with Solid and Liquid Flame Retardants: Comparative Study

**DOI:** 10.3390/polym17141977

**Published:** 2025-07-18

**Authors:** Dorota Głowacz-Czerwonka, Patrycja Zakrzewska, Beata Zygmunt-Kowalska, Iwona Zarzyka

**Affiliations:** 1 Department of Organic Chemistry, Rzeszow University of Technology, 35-959 Rzeszow, Poland; izarzyka@prz.edu.pl; 2 Department of Heat Engineering and Environment Protection, AGH University of Krakow, 30-059 Krakow, Poland; trestka@agh.edu.pl (P.Z.); zygmunt@agh.edu.pl (B.Z.-K.)

**Keywords:** fire-retardant composites, polymer stability, combustion behaviour, thermal degradation, construction materials

## Abstract

The thermal properties and flammability of rigid polyurethane foams (RPUFs) containing various flame retardants, including solid (melamine, expanded graphite (EG), Exolit OP 935, ammonium polyphosphate (APP)) and liquid (Roflam B7, Roflam PLO) types, added at 30 wt.% and 60 wt.% by weight have been evaluated. Thermogravimetric analysis (TGA) demonstrated enhanced thermal stability, with the maximum 10% weight loss temperature (292 °C, +34 °C vs. reference) observed for foams containing 60 wt.% Exolit OP 935 and APP. The limiting oxygen index (LOI) test demonstrated the optimal performance for 30 wt.% APP and melamine (26.4 vol.% vs. 18.7 vol.% reference). In the UL-94 test, Exolit OP 935 and APP achieved a V-0 rating. The 60 wt.% Exolit with an EG blend also demonstrated a substantial reduction in heat release rate. These findings underscore the cooperative effects of hybrid flame retardants, thereby supporting their utilization in fire-safe RPUFs for construction and transport.

## 1. Introduction

Rigid polyurethane foam (RPUF) is a polymer-based foam containing carbamate groups in its main chain [1]. It possesses a closed-cell structure, low thermal conductivity, low moisture permeability, high specific strength, solvent resistance, good adhesion, and low density [2,3,4]. Density is a pivotal factor influencing the properties of polyurethane materials. Research has demonstrated a direct correlation between density and the mechanical strength, modulus, and energy absorption of RPUF, thereby determining its range of applications [5]. Low-density PUR is commonly used in cushions and mattresses, while higher-density variants are used as structural materials, energy-absorbing components, and shock dampers [6,7]. Notably, recent advancements have led to the utilisation of RPUFs in sealing nuclear weapon stockpiles, underscoring their versatility and potential for critical applications. With a growing focus on energy efficiency and environmental protection, RPUFs are widely used in construction as thermal insulation for windows, walls, roofs, and structural elements [8].

However, a salient disadvantage of RPUFs is their suboptimal thermal stability and considerable flammability [9]. This poses a significant risk to both human safety and the environment, as the combustion of RPUFs releases toxic gases such as NH_3_, CO, and HCN, which are hazardous to human health [10]. Consequently, all materials utilised in construction must adhere to the stipulated fire safety standards. According to EN 13501-1 [11], building materials, including insulation, should belong to flammability class A1 (non-combustible) or A2 (limited combustibility) [12]. In addition, RPUFs, which are also employed as thermal barriers, are required to demonstrate adequate thermal stability.

RPUFs are highly flammable materials with a low limiting oxygen index (LOI) of around 18%, making them susceptible to rapid and intense combustion, often accompanied by the release of toxic gases [13]. In fire conditions, they burn rapidly and intensively, releasing toxic gases. To enhance their fire safety, flame retardants (FRs) are frequently incorporated during the manufacturing process. These flame retardants can be categorised into two main types: additives and reactives [14]. Additive FRs are more straightforward to integrate and are cost-effective, but they have the potential to adversely affect the material’s properties. Conversely, reactive FRs undergo a chemical bond within the polymer matrix during the synthesis process, providing enhanced flame retardation and long-term stability [15]. However, they are more complex and expensive to utilise. In recent years, there has been a discernible shift away from halogen-based FRs, owing to their propensity to generate toxic combustion by-products. Consequently, halogen-free alternatives such as expanded graphite, ammonium polyphosphate, melamine derivatives, and phosphorus–nitrogen compounds are gaining popularity due to their lower toxicity and smoke emission [16,17,18]. The efficiency of FRs is contingent on their chemical structure and the mechanism they utilise, which can be physical or chemical, and whether they operate in the gas or condensed phase. These mechanisms may inhibit combustion by diluting flammable gases, absorbing heat, or forming a protective char layer. The primary function of FRs is to disrupt the self-sustaining combustion process as expeditiously as possible [19,20]. In recent years, significant advances have been witnessed in the field of enhancing the flame retardancy of polymeric materials through the utilisation of modified organophosphorus compounds, nanocomposites, and hybrid fire retardant systems. An exemplar of an innovative approach is the utilisation of melamine-modified zinc phytate, which has exhibited not only high efficiency in reducing the flammability of silicone rubber but also significant suppression of smoke emissions [21]. In the context of polyurethane foams, the implementation of analogous modification strategies—particularly the combination of solid and liquid retardants—has been shown to result in a synergistic enhancement of flame-retardant properties, while concurrently ensuring the maintenance of critical mechanical parameters.

The primary objective of this study was to undertake a comprehensive evaluation of the thermal stability and flammability of RPUFs modified with various FRs, both solid and liquid, applied individually and in hybrid systems at different concentrations. A particular emphasis was placed on the assessment of cooperative effects arising from the combination of conventional solid additives, including melamine, expanded graphite, Exolit OP 935, and APP, with liquid retardants such as Roflam B7 and Roflam PLO. This study undertakes a comparative analysis of hybrid FR systems at elevated concentrations, with a particular focus on the 60 wt.% combined additives, a subject that is seldom investigated in the extant literature. Typically, commercial formulations use 10–30 wt.% FRs, as higher levels may affect mechanical properties. This research examines the synergistic interactions between solid and liquid FRs in rigid polyurethane foams (RPUFs), providing new insights into thermal stability, char formation, and flame inhibition mechanisms.

## 2. Materials and Methods

### 2.1. Production of Rigid Polyurethane Foam

The materials used Rokopol RF-551 (polyether polyol, purity ≥ 98%, PCC Rokita), triethylamine (TEA, catalyst, purity ≥ 99%, Sigma-Aldrich, Darmstadt, Germany), Silicone L9600 (surfactant, purity ≥ 98%, Momentive Performance Materials Inc., Waterford, NY, USA), distilled water (used as a chemical blowing agent), and polymeric methylene diphenyl diisocyanate (p-MDI, isocyanate component, purity ≥ 98%, BASF). Rigid polyurethane foam (RPUF) composites were synthesized using a one-step method to ensure a simple and efficient production process. Rokopol RF-551 served as the base polyol and was combined with the listed additives to optimize foam formation and structural properties. TEA accelerated the polymerization reaction, while Silicone L9600 stabilized the foam structure and regulated cell morphology. Water reacted with p-MDI to generate CO_2_, facilitating foam expansion. The p-MDI also reacted with the polyol to form the polyurethane matrix. A detailed composition of the composite formulations, including component ratios, is presented in Table 1.

### 2.2. Flame Retardants Applied to RPUFs

To enhance the flame resistance of the obtained RPUF composites, both liquid and solid flame retardants (antipyrines) were incorporated into the polyurethane matrix at two different types: 30% and 60% by weight. The solid flame retardants included melamine, which acts through endothermic decomposition and gas dilution; expanded graphite, forming an insulating char layer; Exolit, an intumescent additive that swells under heat; and ammonium polyphosphate (APP), a phosphorus-based flame retardant that promotes char formation and releases flame-inhibiting gases. Conversely, the liquid flame retardants encompassed Roflam B7, a brominated phosphate ester that facilitates both vapor-phase and condensed-phase flame inhibition, and Roflam PLO, a phosphorus-based liquid additive that enhances thermal stability and reduces flammability. The compositions and ratios of these FRs within the polyurethane system are detailed in Table 1.

Water is a blowing agent. Its introduction (as a result of the reaction with isocyanate) creates carbon dioxide, which foams the reaction mixture. The optimum (2 wt.%) amount of water was used, analogous to that used in commercial foams. The introduction of a smaller amount of water makes the foaming process more difficult, while a larger amount causes the foams to become more brittle.

### 2.3. Methods

The foaming process was evaluated by determining characteristic times, i.e., cream time, gel time, and tack-free time.

The apparent density of the obtained RPUFs composites was determined in accordance with PN-EN ISO 845:2010 [22] by determining the dimensions of the 30 × 30 × 30 mm shapes using a calliper and examining the weight using an analytical balance.

TGA testing was carried out on selected foam compositions and performed with a thermogravimetric apparatus (Thermogravimetric Analyzer TGA2, Mettler Toledo, Greifensee, Switzerland) (in the temperature range 25–600 °C, using nitrogen as the inert gas—flow rate 50 mL/min). The heating rate was 10 °C/min.

The horizontal combustion test for foams was carried out in accordance with the requirements of PN-EN ISO 3582:2002 [23]. The test was carried out by applying a flame with a diameter of 20 ± 2 mm to the sample for a time of 15 ± 0.5 s, keeping a distance of 10 mm from the edge of the sample. Samples with dimensions of 150 × 50 × 13 mm were placed horizontally in a special holder, and the flame source (burning propane) was applied to their free edge. After the flame was removed, the time of further burning and the length of the charred area were measured. The limiting oxygen index (LOI) was performed in accordance with PN-EN ISO 4589-3:2017-06 [24] using the Concept Fire Testing machine (Concept Controls Inc., Aurora, IL, USA). For this purpose, 10 × 10 × 100 mm shapes were made, which were placed in the machine, then covered with a glass tube and set on fire using a gas burner.

Fire behaviour testing was carried out in accordance with PN-EN ISO 13927:2009 [25] using a cone microcalorimeter (Fire Testing Technology FTT Mass Loss Calorimeter (Fire Testing Technology Ltd. (FTT), East Grinstead, UK)). The 100 × 100 × 20 mm samples were cut and subjected to 25 kW/m^2^ of radiant heat. This test identified the following parameters: HRR (heat release rate), pHRR (peak heat release rate), THR (total heat release), EHC (effective heat of combustion), TTI (time to ignition), TTF (time to flameout), PML (percentage mass loss), MLR (mass loss rate).

Flammability test in a UL-94 chamber according to EN 60695-11-10 [26]. For the test, a 120 × 12.7 × 12.7 mm sample was cut. Then, the measurement was carried out with a gas burner. The result of the test is the classification of the tested material to the flammability class: HB, V-2, V-1, V-0, 5VB, and 5VA.

## 3. Results

### 3.1. Foaming Process of Foams

**Figure 1:**
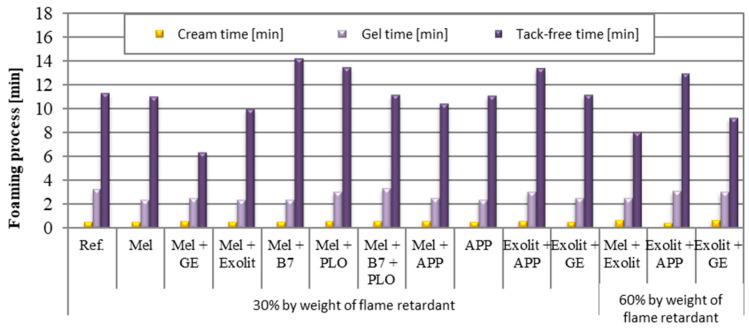
Foaming process of foam composites.

Figure 1 shows the effect of different fire retardants and their concentrations on the foaming process of foam composites. The foaming process considers three stages: creme time, gel time, and tack-free time.

For the cream time, which marks the onset of foaming, this time is similar for all the samples tested (approximately 0.5 min), regardless of the type or concentration of flame retardant used. This suggests that the additives have no significant effect on the initial phase of the foaming process. Gel time for all samples tested range from approximately 2.36 to 3.30 min. Foams containing PLO show longer gel times (3.07–3.33 min) compared to, for example, foams that contain flame retardants in combination with Exolit. This difference is mainly due to the different chemical natures of the additives used. Exolit, as a typical flame retardant [27,28], does not significantly affect the reaction kinetics. In addition, the presence of PLO as a plasticiser can increase the viscosity of the system, slowing down the diffusion of the reactants and consequently increasing the gel time [29]. In the case of the reference sample, the absence of any additives causes the process to proceed with natural, unaccelerated kinetics. However, it should be noted that the observed time differences are relatively small (1–1.5 min), indicating a similar overall effect of the systems tested on the gelation process. The exceptions to this are the APP-containing systems, which clearly shorten the gel time, while the other compositions show similar process characteristics. Analysis of the results showed significant differences in tack-free time values between the samples tested, ranging from approximately 6 to 14 min. The shortest tack-free times (~6 min) were observed in samples containing Mel + GE, which can be attributed to the catalytic effect of GE accelerating the surface curing process. In contrast, the longest times (~14 min) occurred in the reference samples and samples with Mel + Roflam B7, where the lack of catalyst and potential plasticizing effect significantly slowed the loss of surface tack. The composition of the mixture is crucial to the rate of stable surface formation: GE accelerates the process, while APP has the opposite effect.

### 3.2. Physical and Thermal Properties of Foams

Increasing the apparent density of RPUF reduces oxygen diffusion and improves the effectiveness of flame-retardant additives, leading to higher fire resistance. At the same time, a higher mass of combustible material per unit volume can increase total heat emission and increase combustion time [30]. Therefore, the flammability analyses were preceded by an analysis of the effect of flame retardants on apparent density and are summarised in Table 2.

The differences in the apparent density of RPUF foams are due to the type of additives used. Melamine is light (specific density approx. 1.5 g/cm^3^) [31] and foams well, which promotes a more porous structure and thus a lower apparent density of the foam. APP, on the other hand, is an inorganic additive with a higher specific density (approximately 1.9 g/cm^3^), which increases the proportion of the solid phase and reduces foam expansion during foaming. Tests on the apparent density of foam composites showed differences depending on the additives used. The lowest density was observed for the sample containing melamine alone (65.7 kg/m^3^) and its combination with Exolit (62.1 kg/m^3^), confirming their lightness and effectiveness in forming a porous structure. In contrast, samples with APP, especially at higher concentrations (60%), had a significantly higher density (75.7 kg/m^3^), as a result of the properties of this filler. Results were obtained for samples containing both melamine and APP (73.7 kg/m^3^), where the density was similar to samples with APP alone, suggesting the dominant influence of APP on the foam structure. In contrast, combinations of melamine with other additives, such as PLO or B7, showed intermediate values (64.4–65.9 kg/m^3^), which may indicate their lesser influence on the foaming process compared to APP.

**Figure 2:**
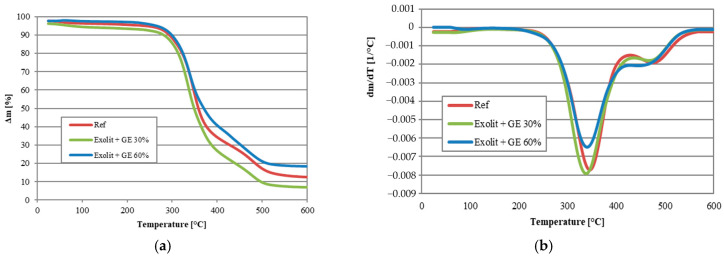
Characteristics of thermal degradation of the foam composites: TG curves (**a**); DTG (**b**) curves.

**Figure 3:**
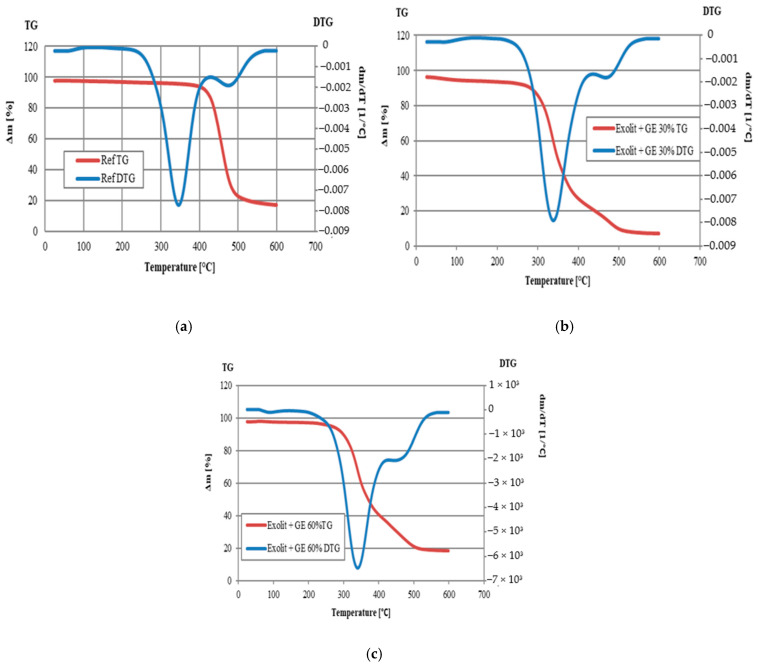
Characteristics of thermal degradation of the foam composites: TG and DTG curves of reference foam (**a**); TG and DTG for foams with 30 wt.% addition of fire retardants (**b**); TG and DTG for foams with 60 wt.% addition of fire retardants (**c**).

As demonstrated in Figure 2 and Figure 3 and Table 3, the thermal stability studies of foam composites provide significant insights into the material’s behaviour at elevated temperatures. The reference sample (Ref) exhibited a standard thermal degradation trajectory, with an initial decomposition temperature (T5%) of 234 °C and a maximum degradation rate at 293 °C (Figure 2a and Figure 3a). The low amount of pyrolysis residue (12.62 wt.% at 600 °C) indicates the predominance of thermal degradation processes, with a low tendency to form stable carbonaceous structures. Analysis of the thermal degradation results of the Exolit + GE 30% sample indicates a decrease in thermal stability compared to both the reference foam and the Exolit + GE 60% sample. The most significant discrepancy was identified from T5%, suggesting that degradation begins at lower temperatures. As shown in this sample, a lower amount of pyrolysis residue was observed at 600 °C (7.00% by weight compared to 12.62% by weight for the reference sample), thus confirming the reduced thermal resistance of the sample in question. The deterioration of thermal properties can be attributed to the inhomogeneous dispersion of flame retardants at a concentration of 30% or suboptimal interactions between the additives and the polyurethane matrix, leading to the formation of local areas of weakened structure. The introduction of fire-resistant modifiers at a concentration of 60 wt.% resulted in a substantial alteration of the thermal degradation characteristics. The Exolit + GE (30 wt.%) sample exhibited a decrease in the onset temperature of degradation to 110 °C (Table 3), which may indicate an accelerated activation of fire-resistant mechanisms. Concurrently, a decline in the residue (7.00 wt.%) was observed, suggesting a divergent mechanism of action in comparison to higher concentrations. The most compelling results were obtained for samples containing 60 wt.% modifiers. The Exolit + GE composite (60 wt.%) was characterized by an increased degradation onset temperature (263 °C) and a significantly higher amount of pyrolysis residue (18.56 wt.%). This observation, when considered in conjunction with the shift of peaks in the DTG curves toward higher temperatures (Figure 3c), suggests the formation of a stable protective layer that effectively slows further thermal degradation.

A study of the multi-stage nature of DTG curves was conducted to determine the effect of modifiers on the degradation mechanism. Primarily two degradation stages were observed in all samples tested, related to the degradation of soft and hard foam segments.

The TGA results are consistent with the mechanism of action of phosphor modifiers, which undergo transformations at higher temperatures that lead to the formation of phosphate layers that limit mass and heat transfer. The combination of Exolit and GE at a concentration of 60 wt.% proved to be particularly effective, with both components increasing thermal stability and resulting in a significantly higher residual amount. This observation confirmed the formation of an effective thermal barrier.

The course of the curves aligns with the observations recorded by other scholars. In the initial phase, there is degradation of the rigid segments of the RPUFs structure, which is associated with the rupture of urethane bonds. In the second stage, the degradation of the soft segments of the RPUF polyol-based structure occurs, leading to the formation of certain types of aliphatic ethers or alcohols [32,33].

### 3.3. Foam Flammability Tests

**Figure 4:**
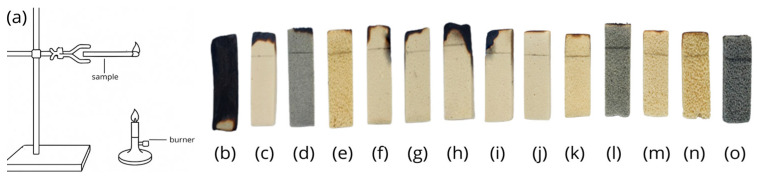
Flammability test: test stand (**a**); Ref (**b**); Mel (**c**); Mel + GE (**d**); Mel + Exolit (**e**); Mel + B7 (**f**); Mel + PLO (**g**); Mel + B7 + PLO (**h**); Mel + APP (**i**); APP (**j**); Exolit + APP (**k**); Exolit + GE (**l**); Mel + Exolit (60 wt.%) (**m**); Exolit + APP (60%) (**n**); Exolit + GE (60 wt.%) (**o**).

**Figure 5:**
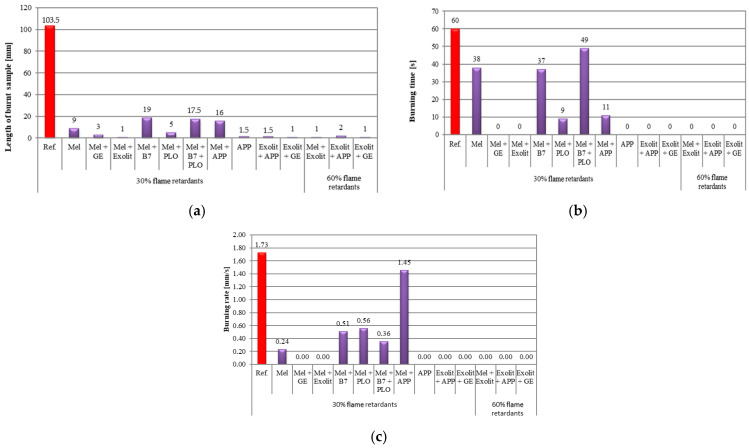
Flammability test results: length of combustible sample (**a**); burning time (**b**); burning rate (**c**).

The flammability test, illustrated in Figure 4 and Figure 5, was conceived with the objective of evaluating the behaviour of diverse materials when subjected to controlled combustion. The test stand depicted in Figure 4a was utilized for conducting the tests, while the subsequent images in Figure 4b–o illustrate various samples, including the reference material (Ref) and modifications of the base material (Mel) with additives such as GE, Exolit, B7, FLQ, and APP, as well as variants with elevated concentrations of planarity-reducing agents (60 wt.%).

The horizontal burning test results presented in Figure 5 include three key parameters: length of the burned sample (a), burning time (b), and burning rate (c). The analysis of these data facilitates an assessment of the effect of individual modifications on the fire properties of the tested materials.

As a point of comparison, a reference sample was used, where the melamine-only sample functioned as the base variant for later changes. The analysis of the results indicated that the Mel sample exhibited moderate flammability, thereby establishing a fundamental baseline for the subsequent evaluation of the efficacy of various modifying additives. In the case of samples containing the Exolit additive (Figure 4e,m,n), a marked reduction in combustion rate was observed in comparison to both the reference sample and pure Melamine. This effect was particularly evident in the sample with the higher concentration (60 wt.%), where the burning time was equal to 0 s, as was the length of the burned sample. This finding suggests that Exolit functions as an effective flame retardant, likely by forming a stable protective layer during combustion that limits oxygen access and slows the thermal decomposition of the material. Furthermore, combinations involving Mel + APP (Figure 4i) demonstrated inferior fire resistance. It was observed that these samples exhibited a higher burning rate and longer burning time in comparison to the Mel sample. Furthermore, intriguing outcomes were attained for specimens comprising the GE additive (Figure 4d,l,o). In such cases, an intermediate performance was observed between Exolit and APP, which may indicate a different mechanism of action of this modifier. Other researchers have reported that foams that do not contain flame retardants have a combustion time of 32–47 s, and the length of the burned sample varies from 62 to 89 mm [34].

As demonstrated in Table 4, which presents the results of the LOI tests, the materials that were examined were found to have various levels of fire resistance. The LOI is a critical metric for evaluating the fire resistance properties of materials, as it quantifies the minimum concentration of oxygen in the nitrogen mixture required for spontaneous combustion [35].

The Ref sample exhibited the lowest LOI value (18.70 vol.%), which is characteristic of flammable materials. Mel foam exhibited a marginally higher value of 21.00 vol.%, indicative of its moderate fire resistance. The introduction of the GE additive (Mel + GE) resulted in a slight improvement of this parameter (21.50 vol.%), while the Exolit additive (Mel + Exolit) caused a more pronounced increase in LOI to 22.40 vol.%, thus confirming its effectiveness as a flame retardant. The most significant effects were observed with combinations of different additives. The Exolit + GE sample attained an LOI value of 25.10%, indicating a substantial synergistic effect of the two modifiers. Samples with higher concentrations (60%) demonstrated enhanced flame retardant properties, with the Exolit + GE (60 wt.%) composition exhibiting a particularly noteworthy result of 29.80 vol.%. This value indicates that the material requires an oxygen content of approximately 30 vol.% in the atmosphere to sustain combustion, thus classifying it as flame retardant. As is evidenced by the extant literature, rigid polyurethane foams typically exhibit this parameter within the range of 21–22 vol.% [36,37].

**Figure 6:**
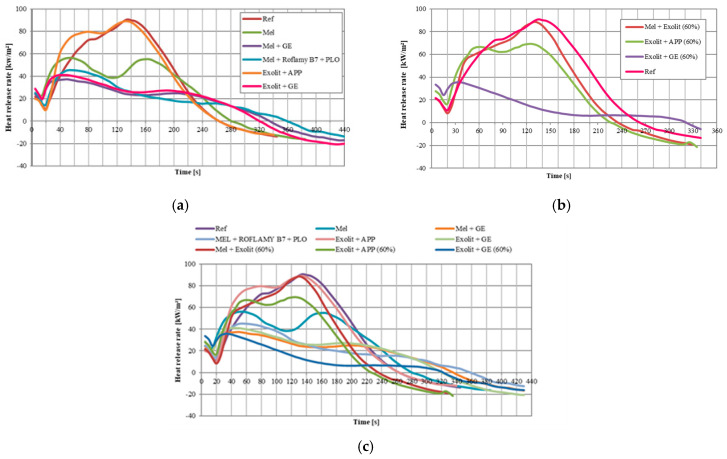
Rate of heat release for samples containing 30% FRs (**a**), samples containing 60% FRs (**b**), and all samples (**c**).

Tests conducted using a cone calorimeter revealed significant differences in the fire behaviour of the materials tested. The results shown in Table 5 and Figure 6 reflect the behaviour of the sample during a realistic fire [38].

An analysis of the combustion parameters indicates that the Ref sample reached the highest values of peak heat release (pHRR = 90.76 kW/m^2^) and total heat release (THR = 13.00 MJ/m^2^). In the case of Mel sample, a significant reduction in these parameters was observed (pHRR = 56.19 kW/m^2^, THR = 10.70 MJ/m^2^), indicating its superior fire resistance compared to the reference material. It has been demonstrated that compositions containing a combination of Exolit with GE are the most effective fire-resistant systems. The Exolit + GE sample demonstrated remarkably low pHRR (35.55 kW/m^2^) and THR (4.60 MJ/m^2^), exhibiting a 61% and 65% decrease, respectively, in comparison to the reference sample. Furthermore, it exhibited the lowest mass loss rate (MLR = 0.021 g/s) and a substantial reduction in effective heat of combustion (EHC = 5.87 MJ/kg). These results provide robust confirmation of the effectiveness of this combination of modifiers in reducing flammability. The Mel + B7 + PLO sample had a high fire resistance (pHRR = 45.33 kW/m^2^, THR = 7.90 MJ/m^2^) despite the relatively lower modifier concentration (30 wt.%). At the same time, this sample had the longest time to ignition (TTI = 12 s) and the longest total burning time (TTF = 400 s).

The following observations are derived from the graphs in Figure 6. For samples containing 30 wt.% modifiers (Figure 6a), it was possible to observe significant differences in the shape of the curves. Samples with GE additives had more stable and controlled combustion compared to other samples. In the case of samples with a higher modifier concentration (60 wt.%, Figure 6b), the Exolit + GE composition had the lowest flammability values obtained in cone calorimeter. In accordance with the guidelines stipulated within the construction industry regarding thermal insulation materials, the PHRR of such materials is required to be less than 300 [kW/m^2^] [39,40].

### 3.4. UL-94 Flammability Test in a Chamber

Flammability testing was conducted in the UL-94 chamber for a composition of Exolit and expanded graphite, with a flame-retardant ratio of 60% by weight. The composition in question was selected for further analysis based on its satisfactory results in other fire resistance tests. The result of this test is an indication of the tendency of the material to extinguish or spread flame upon ignition.

During the experiment, the foam did not ignite; instead, it merely osmolized and formed a layer of char. Consequently, it was classified in the flammability class V-0. This class is the most advanced, as it is indicative of the fact that the material which has been subjected to testing does not ignite under general conditions.

The composition with Exolit and expanded graphite, at a 60 wt.% by weight ratio of flame retardant to polyol, exhibits optimal fire properties. The composition is characterized by an oxygen index of 29.8 vol.%, representing its highest level of flammability. This is categorized as V-0, indicating its designation as a non-flammable material. In the horizontal test, the rate of heat release was found to be the lowest. This composition did not ignite but was osmolated. Furthermore, the weight loss was negligible.

## 4. Conclusions

The successful production of rigid polyurethane foam composites with reduced flammability can be achieved through the utilization of a combination of solid and liquid flame retardants.

The results of physical property tests conducted demonstrated that the apparent density of the foam composites obtained fell within the range of 60 to 80 kg/m^3^. A study of the thermal properties of TGA demonstrated that the onset of decomposition occurred at 260 °C, with the most rapid weight loss occurring in the range of 330–340 °C. A study of the fire properties of the foam composites produced was carried out, which included determining the oxygen index and performing a horizontal test. It showed that the composite, which was characterised by 60 wt.% of a mixture of flame retardants (Exolit and expanded graphite), exhibited optimal fire properties. The foam did not ignite and had an oxygen index of 29.8 vol%, allowing it to be classified as a non-combustible material. In addition, fire parameters were investigated using a cone microcalorimeter, of which the HRR parameter is one of the most important. The composition obtained with 60 wt.% Exolit and expanded graphite showed the lowest heat release rate (35.55 kW/m^2^). A UL-94 V test was also carried out, which confirmed the non-flammability of the tested composition and allowed it to be classified in the V-0 flammability class.

The research indicates that a combination of flame retardants, namely Exolit and expanded graphite, is the most effective method of modifying the foam to make it non-combustible. Moreover, the research presented in this paper demonstrates an innovative method to produce non-combustible thermal insulation materials.

## Figures and Tables

**Table 1:** Components and FR content of RPUF composites.

Sample	Description	Substrates							
		Rokopol RF 551	Flame Retardant [g]			TEA [g]	L900 [g]	Water [%]	MDI [g]
			1	2	3				
Ref	Reference foam	100	0	0	0	0.66	0.14	2	197
Mel	Foam with 30 wt.% melamine added	100	30	0	0	0.66	0.14	2	197
Mel + GE	Foam containing 15 wt.% melamine and 15 wt.% expanded graphite 290	100	15	15	0	0.66	0.14	2	197
Mel + Exolit	Foam containing 15 wt.% melamine and 15 wt.% Exolit OP 935	100	15	15	0	0.66	0.14	2	197
Mel + B7	Foam containing 15 wt.% melamine and 15 wt.% Roflam B7	100	15	15	0	0.66	0.14	2	197
Mel + PLO	Foam containing 15 wt.% melamine and 15 wt.% Roflam PLO	100	15	15	0	0.66	0.14	2	197
Mel + B7 + PLO	Foam containing 10 wt.% added melamine, 10 wt.% Roflam B7, and 10% Roflam PLO	100	10	10	10	0.66	0.14	2	197
Mel + APP	Foam containing 15 wt.% melamine and 15 wt.% ammonium polyphosphate	100	15	15	0	0.66	0.14	2	197
APP	Foam containing 15 wt.% ammonium polyphosphate	100	30	0	0	0.66	0.14	2	197
Exolit + APP	Foam containing 15 wt.% of Exolit OP 935 and 15 wt.% of ammonium polyphosphate	100	15	15	0	0.66	0.14	2	197
Exolit + GE	Foam containing 15 wt.% Exolit OP 935 and 15 wt.% expanded graphite EG 290	100	15	15	0	0.66	0.14	2	197
Mel + Exolit (60 wt.%)	Foam with 30 wt.% melamine and 30 wt.% Exolit OP 935	100	30	30	0	0.66	0.14	2	197
Exolit + APP (60 wt.%)	Foam containing 30 wt.% Exolit OP 935 and 30 wt.% ammonium polyphosphate	100	30	30	0	0.66	0.14	2	197
Exolit + GE (60 wt.%)	Foam containing 30 wt.% Exolit OP 935 and 30 wt.% expanded graphite EG 290	100	30	30	0	0.66	0.14	2	197

**Table 2:** Apparent density of foam composites (standard deviation (SD) 0.21–0.33).

Sample	Apparent Density [kg·m^−3^]
Ref	64.2
Mel	65.7
Mel + GE	67.8
Mel + Exolit	62.1
Mel + B7	65.4
Mel + PLO	65.9
Mel + B7 + PLO	64.4
Mel + APP	73.7
APP	71.5
Exolit + APP	69.1
Exolit + GE	66.8
Mel + Exolit (60%)	75.3
Exolit + APP (60%)	75.7
Exolit + GE (60%)	73.2

**Table 3:** Characteristics of thermal degradation of the foam composites.

Sample	T_5%_ [°C]	T_10%_ [°C]	T_15%_ [°C]	T_20%_ [°C]	T_50%_ [°C]	Residue at 600 °C [wt.%]
Ref	234	293	310	321	358	12.62
Exolit + GE 30%	110	282	302	313	348	7.00
Exolit + GE 60%	263	298	313	324	370	18.56

**Table 4:** Results for the LOI standard deviation (SD) 0.08–0.15.

Sample	LOI [vol.%]	
Ref	18.70	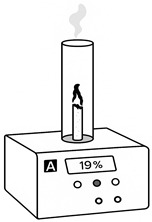
Mel	21.00	
Mel + GE	21.50	
Mel + Exolit	22.40	
Mel + B7	20.30	
Mel + PLO	21.00	
Mel + B7 + PLO	21.20	
Mel + APP	20.70	
APP	21.20	
Exolit + APP	21.70	
Exolit + GE	25.10	
Mel + Exolit (60 wt.%)	24.20	
Exolit + APP (60 wt.%)	23.90	
Exolit + GE (60 wt.%)	29.80	

**Table 5:** Cone calorimeter results for foam composites.

Sample	HRR [kW/m^2^]	pHRR [kW/m^2^]	THR [MJ/m^2^]	EHC [MJ/kg]	TTI [s]	TTF [s]	PLM [wt.%]	MLR [g/s]
Ref	95.00	90.76	13.00	8.05	13	215	82.50	0.070
Mel	40.62	56.19	10.70	9.32	5	261	69.50	0.038
Mel + GE	23.34	37.27	7.70	7.57	6	327	5.30	0.027
Exolit + APP	65.14	88.81	13.50	8.77	13	219	81.40	0.065
Mel + B7 + PLO	19.02	45.33	7.90	6.33	12	400	68.30	0.026
Exolit + GE	25.00	41.01	8.10	7.01	7	322	64.80	0.031
Mel + Exolit (60%)	62.20	88.53	11.0	7.52	14	187	74.00	0.072
Exolit + APP (60%)	55.57	69.39	9.90	7.29	11	186	77.20	0.067
Exolit + GE (60%)	13.98	35.55	4.60	5.87	8	313	39.30	0.021

## Data Availability

The data presented in this study are available on request from the corresponding author.

## Abbreviations

RPUF	rigid polyurethane foam
REF	reference sample
MEL	melamine
EG	expanded graphite
APP	ammonium polyphosphate
TGA	thermogravimetric analysis
UL-94	Underwriters Laboratories 94
LOI	limiting oxygen index
FR	flame retardant
HRR	heat release rate
pHRR	peak heat release rate
EHC	effective heat of combustion
THR	total heat release
TTF	time to flameout
TTI	time to ignition
PLM	percentage mass loss
MLR	mass loss rate
